# Internet benefits are not one-size-fits-all: age and trust shape digital wellbeing gains

**DOI:** 10.3389/fpsyg.2026.1736005

**Published:** 2026-04-10

**Authors:** Yongjun Yu, Siyu Luo, Pei Sun

**Affiliations:** Faculty of Health and Wellness, City University of Macau, Macau, Macao SAR, China

**Keywords:** age moderation, China, digital wellbeing, frequency of internet use, moderated mediation, social interaction, social trust, subjective wellbeing

## Abstract

**Background:**

Although internet use is widespread, its relationship with subjective wellbeing (SWB) remains complex. While previous studies have noted the role of social interaction, less is known about how age and social trust shape this process.

**Methods:**

This study investigates how the frequency of internet use relates to SWB among Chinese adults, focusing on the mediating role of social interaction and the moderating roles of social trust and age. Using data from the 2021 Chinese General Social Survey (*N* = 8,148), we analyzed these relationships through a moderated mediation model.

**Results:**

The results indicated that: (1) frequent internet use was positively associated with social interaction; (2) social interaction fully mediated the link between internet use and SWB; (3) the positive relationship between internet use and social interaction was stronger for individuals with higher social trust, but weaker among older adults; and (4) the indirect association of internet use was most significant for younger individuals and those with higher social trust.

**Discussion:**

These findings suggest that the potential link to wellbeing depends primarily on social interaction, a pathway that varies by individual trust levels and age. The study points to the need for digital policies that consider these demographic and psychological differences.

## 1 Introduction

In the digital era, the internet is deeply integrated into daily life, and its impact on psychological and social wellbeing has become a key focus of academic research (Firth et al., 2024). Existing studies on the relationship among internet use, social interaction, and subjective wellbeing (SWB) have formed a general consensus. On one hand, the internet makes it easier for people to socialize across time and space through social media and instant messaging, helping to expand social networks and increase the frequency of interaction (Bessière et al., 2008). On the other hand, social interaction is a critical source of SWB, as frequent, high-quality social contact is strongly linked to greater wellbeing (Clark et al., 2018; Utz and Breuer, 2017). However, research has also highlighted potential downsides, such as information overload, social comparison, and problematic use, which may undermine wellbeing—particularly among adolescents and young adults (Twenge et al., 2018; Zhao, 2022). Given these mixed findings, the effect of internet use on SWB is not always straightforward, and its underlying mechanisms and

boundary conditions require deeper exploration. Although past research has highlighted the mediating role of social interaction in the link between internet use and SWB (Chen and Zeng, 2022; Liu et al., 2025), less attention has been paid to which individuals are more likely to gain happiness from these online-supported interactions. In other words, the role of moderating factors has not been systematically pieced together. Age, as a key marker of a person's developmental stage, can shape the motivations for using the internet, the quality of social outcomes, and overall wellbeing (Hong et al., 2016; Zhang and Li, 2022; Guo and Ma, 2023; Peng and Hu, 2025). At the same time, social trust—reflecting an individual's confidence in social exchanges—may strengthen or weaken the positive effects of these interactions (Zhang and Li, 2023; Wang and Zhou, 2019; Miao et al., 2025; Zhang and Shan, 2021). Notably, the work of (Zhang and Shan 2021) also established a direct link between internet use and social trust, adding further evidence to the mechanisms at play. While previous studies have extensively examined the connections among internet use, social interaction, social trust, and SWB, there is a lack of research that examines their interactive mechanisms within a unified framework, especially regarding systematic differences based on age. Guided by social support theory and digital divide theory, this study proposes a mediation model where internet use relates to SWB through the channel of social interaction. We introduce social trust as a moderator and also examine how these relationships hold across different age groups. Our goal is to elucidate how these dynamics vary by age in the digital era. Therefore, this study focuses on two main research questions: How is the frequency of internet use associated with SWB through social interaction? And do age and social trust moderate this indirect pathway? By testing a moderated mediation model, we aim to examine the relationships among these variables. This work seeks to contribute to the understanding of the link between digital technology and wellbeing, while providing empirical evidence for age-inclusive digital policies.

## 2 Theoretical underpinnings and hypotheses

### 2.1 Guiding theoretical frameworks

This study's theoretical model is constructed upon an integration of several key frameworks. Social Support Theory provides the foundational structure for understanding the mediating role of social interaction (Cohen and Wills, 1985). Social Cognitive Theory offers a critical perspective for elucidating the moderating role of social trust (Bandura, 1986). Socioemotional Selectivity Theory (SST) serves as the central theory for interpreting the moderating effect of age (Carstensen, 1992). Finally, Digital Divide Theory provides an essential complementary lens for explaining the observed age-related differences (Van Dijk, 2006).

Social Support Theory posits that social support consists of resources obtained through one's social network that promote physical and mental health. It can be categorized into objective support, subjective support, and the degree of support utilization. The theory proposes two pathways through which support relates to SWB: a “main effect model,” which directly fosters feelings of belonging and happiness, and a “buffer model,” which provides protection during stressful situations. In the context of this study, the internet is viewed as an enabling tool. Its use may facilitate the frequency and quality of social interaction, thereby increasing an individual's likelihood of obtaining social support—particularly emotional and companionship support—which in turn is associated with higher SWB (Sun et al., 2023). Therefore, Social Support Theory is the core rationale for arguing the mediating pathway of social interaction.

Social Cognitive Theory emphasizes the concept of triadic reciprocal determinism, where personal cognitive factors, behavior, and the environment interact and shape one another. Social trust, as a stable cognitive belief—a generalized expectation of others' goodwill and reliability—plays a role in an individual's interpretation of and behavioral response to the online social environment. Individuals with high social trust are more inclined to make benign attributions for ambiguous online social cues and engage in proactive social behaviors. This may help them to more easily convert internet use into high-quality social connections and positive emotional experiences. Conversely, individuals with low trust may inhibit their social investment due to heightened risk perception and a defensive psychological stance, thereby potentially diminishing the benefits of internet use (Flanagan and Stout, 2010; Miao et al., 2025).

Socioemotional Selectivity Theory (SST) argues that an individual's choice of social goals changes according to their perceived future time perspective. Young people, possessing an expansive view of the future, tend to focus their internet use on future-oriented goals such as social network expansion and information acquisition. While they face risks such as social overload and internet addiction (Twenge et al., 2018), their higher digital literacy and social motivation enable them to more readily transform online-to-offline social support, potentially benefiting their SWB (Kavetsos and Koutroumpis, 2011). In contrast, older adults, often constrained by a digital skills gap and shrinking social networks, concentrate their internet use on maintaining strong ties and seeking emotional support. Although this usage pattern can provide emotional satisfaction (Cotten et al., 2014), the resulting association with SWB tends to be weaker than that of younger people due to limited usage intensity and social breadth (Chen and Schulz, 2016). Thus, SST reveals the intrinsic mechanism through which age moderates the relationship between internet use and user motivations.

Digital Divide Theory offers an important complementary perspective on these age-related differences. It posits that systematic disparities exist across social groups in terms of internet access, digital skills, and the ability to benefit from online resources. In this study, this framework, combined with life-span developmental theory, helps explain the heterogeneity among age groups. Young adults (digital natives) are proficient internet users but may be more vulnerable to its negative effects. Middle-aged adults (digital immigrants) are relatively better at balancing online and offline social resources. Older adults face the most significant digital skills divide, suggesting their outcomes from internet use may rely more on pre-existing strong ties and their level of social trust (Büchi et al., 2019). This perspective clarifies that the moderating role of age may stem not only from psychological motivations (as per SST) but also from structural inequalities in digital resource access and competence.

Additionally, the Technology Acceptance Model (TAM; Davis, 1989) provides a complementary lens. It posits that perceived ease of use and perceived usefulness are key determinants of technology adoption and effective use. In our context, age may be linked to perceived ease of use (a first-level digital divide), while social trust may relate to the perceived usefulness of online social tools (a second-level divide). Together, they may moderate the likelihood that internet use translates into rewarding social interaction.

### 2.2 The relationship between frequency of internet use and subjective wellbeing

The relationship between frequency of internet use and SWB is a core issue in sociology, psychology, and communication studies, with scholars presenting a rich and complex discussion based on diverse theories and empirical data. Broadly, two opposing explanatory paths dominate the field: one emphasizing the positive impacts of internet use, and the other focusing on its potential negative consequences.

The positive perspective posits a direct positive association between frequency of internet use and SWB. By providing efficient information access, convenient tools for social maintenance, and abundant entertainment options, the internet significantly enhances individuals' social connections and psychological resources (Kraut and Burke, 2015). Especially in unique periods like the post-pandemic era, the internet has become a critical channel for maintaining social functioning and psychological resilience (Liu et al., 2025). Studies have found that for middle-aged and older adults, higher frequency and more diverse use of the internet correlate with higher life satisfaction (Jin et al., 2024). For youth, frequent internet use for learning, work, and socializing benefits their psychological state (Guo et al., 2020), and the intensity of social media use can enhance their SWB by increasing online social support (Hu and Guo, 2021). Furthermore, research on rural residents shows that increased internet use, for both productive and lifestyle purposes, boosts their happiness, with lifestyle-related use having a more pronounced effect (Zhang and Liu, 2020). These findings collectively support the mobilization perspective or the view of digital engagement benefits, which holds that the internet, as a technological tool, effectively promotes interpersonal interaction, social capital accumulation, and experience sharing, thereby generating a net positive effect on residents' SWB (Du and Wang, 2020).

It is important to note that the measure of “frequency of internet use” in this study, derived from the CGSS survey, captures the overall frequency of general internet access (including mobile access). While this aggregated measure is widely used in large-scale social surveys to assess digital engagement (e.g., Zhu et al., 2024), it does not differentiate between specific types of online activities (e.g., social networking, information seeking, entertainment). Our focus is thus on the general frequency of use as a precursor to social opportunities.

Conversely, the negative perspective is encapsulated by the time displacement hypothesis (Nie and Hillygus, 2002). This view posits that excessive time spent online may foster interpersonal and societal alienation, as offline social interactions are displaced by online engagements. Evidence from the Chinese context supports this: such substitution can diminish young people's enthusiasm for participating in political and social activities (Zeng, 2013), or reduce the “presence space” for older adults (Du and Wang, 2020). Furthermore, exposure to online misinformation may exacerbate social tensions. When confronted with an overwhelming volume of online information, individuals may experience information overload, cognitive fragmentation, and decision fatigue, thereby inducing psychological strain (Hughes Jr and Hans, 2001). Additionally, online activities can encroach upon time for face-to-face social interaction, reducing opportunities to build deep trust. Adolescents, in particular, may experience heightened anxiety from excessive investment in maintaining social media presence (Zhao, 2022). Immersion in virtual environments may also contribute to internet addiction and a regression in social skills. Persistent social comparison—for instance, with the curated “perfect lives” presented by others—can engender feelings of inferiority and relative deprivation, ultimately reducing life satisfaction (Chen et al., 2021). Empirical studies further indicate that over-reliance on the internet can weaken civic engagement, increase loneliness, and undermine SWB (Sabatini and Sarracino, 2017; Zhang et al., 2020).

In summary, the relationship between frequency of internet use and SWB is not a simple linear correlation. Its direction and strength are complexly moderated by multiple factors, including usage patterns, context (e.g., work, study, entertainment), and user characteristics (e.g., age, personality). Although evidence exists for decreased wellbeing due to problematic use, the majority of research suggests that within a moderate range, the advantages of the internet—efficiency, convenience, and openness—bring significant benefits to residents' information access, interpersonal communication, and leisure. Based on the literature reviewed, the following hypothesis is proposed:

**H1:** Frequency of internet use is positively associated with subjective wellbeing.

### 2.3 The mediating role of social interaction

Internet use may facilitat the frequency of social interaction through multiple mechanisms, which in turn may relate to an individual's SWB. On one hand, the internet provides diverse communication platforms (e.g., instant messaging, social media, video calls), enabling individuals to stay in touch with others more conveniently (Wellman et al., 2001). On the other hand, it helps reduce the spatio-temporal constraints and costs of communication, which supports the maintenance of long-distance social relationships (Huang, 2010). Concurrently, the internet connects like-minded individuals through interest-based communities and forums, providing common topics and sustained motivation for social interaction (Lambert et al., 2013). Meta-analytic evidence from longitudinal studies suggests positive associations between internet use and social interaction (Shklovski et al., 2006).

Social Support Theory (Cohen and Wills, 1985) offers a systematic explanation for this mediating pathway. The theory posits that social interaction bolsters SWB by providing multidimensional support resources, such as emotional, informational, and companionship support. This is corroborated by meta-analyses. For instance, (Pinquart and Sörensen 2000)'s meta-analysis of 286 studies found that social network quality is positively associated with subjective wellbeing, with the quality of social contacts showing stronger associations than the quantity. Similarly, (Song and Fan 2013)'s meta-analysis in Chinese samples found that social support is positively associated with subjective wellbeing, life satisfaction, and positive affect. This support is theorized to operate jointly through a “main effect path” (directly cultivating a sense of belonging and self-worth, thus enhancing life satisfaction) and a “buffer effect path” (alleviating the psychological impact of negative events). This theoretical framework is especially relevant in the context of internet use. We focus on the frequency (structural aspect) of social interaction as it represents accessible opportunities for support and is a robust predictor in population studies. Longitudinal evidence suggests that internet use predicts increased subsequent social contact frequency (Yu et al., 2021; Toh and Khoo, 2025), providing a rationale for testing it as a mediator. The qualitative aspect (e.g., perceived support) is an important avenue for future research.

As a core pathway for satisfying the need for belonging and acquiring emotional support, social interaction is a key predictor of SWB (Myers, 2000). By transcending temporal and spatial constraints, the internet affords diverse communication channels for different age groups: young people rely on social media to maintain high-frequency interactions, middle-aged individuals use communication tools to coordinate family and work relationships, and older adults strengthen family emotional bonds through video calls (Wellman et al., 2001). Frequent social interaction not only increases opportunities to obtain social support (Cohen and Wills, 1985), but also fosters a sense of belonging by strengthening social bonds (Baumeister and Leary, 1995) and directly promotes positive emotions through the sharing of positive experiences (Gable et al., 2018).

Accordingly, the following hypothesis is proposed.

**H2:** Social interaction mediates the relationship between frequency of internet use and subjective wellbeing.

### 2.4 The moderating role of social trust

Social trust, defined as an individual's generalized belief in the reliability and goodwill of others (Wang and Zhou, 2019), plays a key moderating role in the process by which internet use is converted into social interaction. According to Social Cognitive Theory, an individual's cognitive assessment of the social environment significantly influences their behavioral patterns (Bandura, 1986). In the context of internet use, social trust conditions how individuals interpret online social cues and whether they are willing to engage in online-to-offline social activities.

Individuals with high social trust are more inclined to believe in the goodwill of their online interaction partners and are thus more willing to initiate or participate in online social activities. This positive orientation enables them to more effectively transform internet use into actual social connections—both online and offline (Lewicki et al., 1998). In contrast, individuals with low social trust may adopt a defensive stance due to heightened risk perception (e.g., concerns about privacy breaches or online fraud), reducing their willingness to engage in social interaction even when they use the internet frequently.

Empirical evidence supports this moderating mechanism. (Flanagan and Stout 2010) found that among adolescents with high levels of social trust, the positive correlation between social networking site use and offline social frequency was stronger, indicating that high-trust individuals are better able to convert online interactions into real social capital. Similarly, research on older adults by (Cotten et al. 2012) revealed that those with high social trust are more likely to establish social connections via the internet, while low-trust individuals may limit their online social engagement due to security concerns.

The moderating effect of social trust operates through two primary mechanisms: *cognitive filtering* and *risk perception regulation*. Individuals with high trust tend to focus on positive information online (e.g., supportive feedback) while filtering out negative content (e.g., online conflicts), thereby facilitating the conversion of internet use into rewarding social interaction. Conversely, individuals with low trust have a stronger perception of online risks, and the anxiety they experience may inhibit their social engagement, weakening the link between internet use and social interaction.

Based on this, the following hypothesis is proposed:

**H3:** Social trust moderates the relationship between frequency of internet use and social interaction, such that the positive effect of internet use on social interaction is stronger among individuals with higher social trust.

### 2.5 The moderating role of age

Socioemotional Selectivity Theory (SST) provides the key theoretical framework for understanding the moderating role of age in the relationship between internet use and social interaction (Carstensen, 1992). The theory posits that an individual's choice of social goals undergoes systematic changes with age. Young people tend to focus on obtaining information and expanding their social networks, pursuing future-oriented social goals. In contrast, older adults are more concerned with emotional satisfaction and maintaining existing intimate relationships, with their social motivations showing clear present-oriented and emotional characteristics (Carstensen, 2006; Carstensen et al., 1999).

These age-graded motivational differences are also reflected in the types of online activities individuals engage in. Younger adults, with their future-oriented perspective, are more likely to use the internet for information seeking, social networking expansion, and instrumental purposes. In contrast, older adults, with their present-oriented and emotionally-focused goals, tend to use the internet primarily for maintaining close relationships, seeking emotional support, and accessing health information (Toh and Khoo, 2025).

Building on SST and digital divide theory, we argue that age moderates not only the motivation for internet use but also the *efficiency* with which internet use is converted into actual social interaction. Younger adults, characterized by higher digital literacy and future-oriented social goals, can more effectively translate internet use into expanded social activities—both online and offline. In contrast, older adults may face constraints due to limited digital skills and shrinking social networks; even when they use the internet, they may be less able to convert that usage into frequent social interaction (Büchi et al., 2019; Hargittai, 2002).

Based on these theoretical arguments, we posit:

**H4:** Age moderates the relationship between frequency of internet use and social interaction, such that the positive effect of internet use on social interaction is stronger among younger adults than among older adults.

### 2.6 Research hypotheses

Building on the theoretical integration outlined in Section 2.1, this study proposes a moderated mediation model to examine how, when, and for whom internet use frequency is associated with subjective wellbeing. The model tests the following four hypotheses:

- **H1:** Frequency of internet use is positively associated with subjective wellbeing.
- **H2:** Social interaction mediates the relationship between frequency of internet use and subjective wellbeing.
- **H3:** Social trust moderates the relationship between frequency of internet use and social interaction, such that the positive effect is stronger among individuals with higher social trust.
- **H4:** Age moderates the relationship between frequency of internet use and social interaction, such that the positive effect is stronger among younger adults than among older adults.

**Figure 1:**
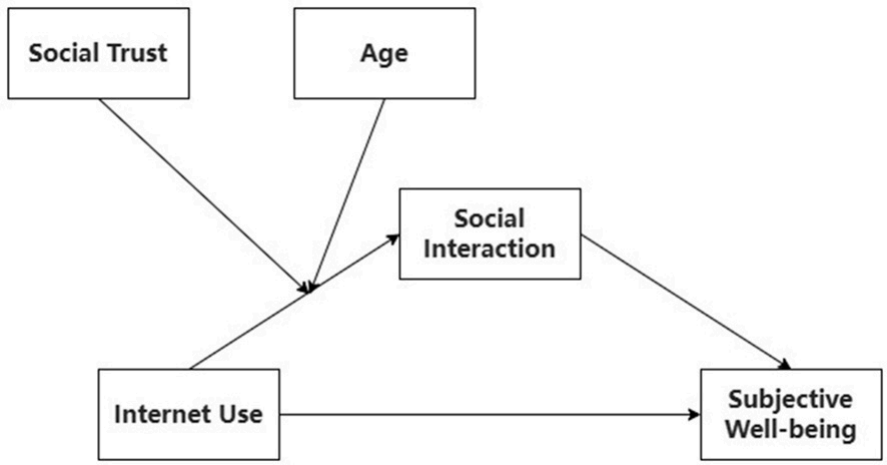
Conceptual model.

Figure 1 presents the conceptual model integrating these hypotheses.

## 3 Materials and methods

### 3.1 Data source

The data for this study were derived from the 2021 edition of the Chinese General Social Survey (CGSS). The CGSS is a nationally representative, continuous survey project administered by the China Survey and Data Center at Renmin University of China. The 2021 wave collected a total of 8,148 samples. The data were obtained from the official National Survey Research Center (NSRC) platform (http://cgss.ruc.edu.cn).

We extend our gratitude to the China Survey and Data Center for providing the data. The analyses and interpretations presented in this paper are solely the responsibility of the author.

### 3.2 Variable description

This study first examines the differences in perceived subjective wellbeing (SWB) among individuals with varying frequencies of internet use. It then investigates the mediating role of social interaction and the moderating roles of social trust and age. Consequently, the primary independent variable is frequency of internet use, and the dependent variable is SWB. Social interaction serves as the mediator, with social trust and age as moderators. Key demographic variables, including gender, years of education, marital status, and household registration (*hukou*), were included as controls. For analytical purposes, gender, marital status, and residence were coded as dummy variables: 0 (female, unmarried, rural) and 1 (male, married, urban).

#### 3.2.1 Dependent variable: subjective wellbeing (SWB)

In line with extensive previous research that has validated the reliability of a single-item measure for SWB (Luo and Wang, 2024; Tian et al., 2024; Li et al., 2024; Wang et al., 2023), we used the response to question A36 from the CGSS questionnaire: “All things considered, how happy would you say you are with your life?” The response was measured on a 5-point Likert scale, ranging from 1 (“Very unhappy”) to 5 (“Very happy”).

#### 3.2.2 Independent variable: frequency of internet use

The independent variable was measured using the item “Your use of the internet (including mobile internet) in the past year.” Respondents reported their frequency of internet use on a 5-point scale, where a higher value indicates greater frequency (Zhu et al., 2024). The specific coding is detailed in Table 1.

**Table 1:** Description of variables and measurements.

Variable	Original question/description	Coding
Gender	Recorded by the interviewer.	0 = Female; 1 = Male
Marital status	What is your current marital status?	0 = Unmarried; 1 = Married
Residence	Indicates the respondent's residential area type.	0 = Rural; 1 = Urban^a^
Years of education	What is your highest level of education?	See conversion note^b^
Frequency of internet use	In the past year, how often did you use the Internet (including mobile access)?	1 = Never; 5 = Very frequently^c^
Subjective wellbeing	Generally speaking, do you feel your life is happy?	1 = Very unhappy; 5 = Very happy^c^
Social interaction frequency	Composite variable based on four items asking about the frequency of social activities.^d^	See original scale^d^
Social trust	Generally speaking, do you think most people in society can be trusted?	1 = Strongly disagree; 5 = Strongly agree^c^
Age	Calculated based on the respondent's year of birth.	Continuous variable

^a^Original text specified “Villagers' committee” for Rural and “Residents' committee” for Urban.

^b^The categorical variable for education was converted into years of schooling as follows: 0 = No formal education; 2 = Private tutoring/Literacy class; 6 = Primary school; 9 = Junior high school; 12 = High school/Secondary specialized/Technical school; 15 = Junior college; 16 = Bachelor's degree; 19 = Postgraduate and above.

^c^This is a 5-point Likert scale. Coding shows the anchor points.

^d^A composite variable from four items (socializing with relatives, friends, neighbors, other friends). Each item was measured on a scale: 1 = Never; 2 = A few times a year or less; 3 = A few times a month; 4 = Once or twice a week; 5 = Almost every day.

#### 3.2.3 Mediating variable: social interaction

Social interaction refers to the interpersonal activities through which individuals engage with relatives, friends, neighbors, and acquaintances, forming and maintaining social relationships. In this study, social interaction primarily refers to the positive interpersonal relationships formed through mutual interaction and joint participation in social activities with relatives, friends, and neighbors.

Following prior studies (Luo and Wang, 2024; Mai and Du, 2024), social interaction was measured using a composite scale constructed from four items assessing the frequency of social activities with relatives, friends, neighbors, and other acquaintances in the past year. Response options ranged from 1 (“Never”) to 5 (“Almost daily”). Although the survey items do not explicitly exclude online interaction, their wording—referring to “getting together,” “visiting,” and “doing activities together”—strongly implies co-presence in physical space within the Chinese linguistic context. We therefore interpret this measure as a core indicator of offline social participation facilitated.

This composite scale assesses the overall frequency of social activities without distinguishing between interaction modalities (e.g., one-to-one vs. one-to-many communication) or their emotional valence. We focus on the structural aspect of social resources (i.e., contact frequency) rather than the qualitative aspect (e.g., perceived social support). The qualitative dimension of social interaction remains an important avenue for future research.

Exploratory factor analysis (EFA) was conducted to assess the scale's dimensionality. The Kaiser-Meyer-Olkin (KMO) measure of sampling adequacy was 0.63, which is considered acceptable for factor analysis with this sample size. A single-factor solution emerged, explaining 37% of the variance. All factor loadings exceeded the 0.40 threshold (range = 0.44 to 0.75, *M* = 0.59), and thus all items were retained for the composite score (Cheung et al., 2024). The scale demonstrated acceptable internal consistency (Cronbach's α = 0.66; Taber, 2018).

#### 3.2.4 Moderating variables: social trust and age

Social Trust: Consistent with common practice in the literature (Lu and Wei, 2023; Yan and Song, 2024), social trust was measured using a single item: “Generally speaking, would you say that most people in society can be trusted?” Responses were captured on a 5-point scale, from 1 (“Strongly disagree”) to 5 (“Strongly agree”), where higher scores indicate a greater level of social trust.

Age: Respondents' age was calculated by subtracting their self-reported year of birth from the survey year (2021).

#### 3.2.5 Control variables

The control variables included gender, marital status, household registration (*hukou*), and years of education. These demographic variables were self-reported by the respondents in the questionnaire.

### 3.3 Data analysis strategy

The statistical analyses were designed to test the moderated mediation model depicted in Figure 1. Following initial data cleaning in SPSS 26.0, all subsequent analyses were conducted in R (version 4.4.3) (R Core Team, 2025). To probe and visualize the significant interaction effects from the model, simple slope analysis was performed and relevant plots were generated using the ggplot2 package (Wickham, 2016).

#### 3.3.1 Common method bias and missing data handling

First, Harman's single-factor test was conducted to assess potential common method bias (Podsakoff et al., 2003). As recommended by (Allison 2009), a logistic regression analysis was performed to formally test the MCAR assumption. To address missing values, we employed multiple imputation (MI) (Baraldi and Enders, 2010; Rubin, 1976; Van Buuren and Van Buuren, 2012; Zhu et al., 2018; Li et al., 2017) using the mice package (van Buuren and Groothuis-Oudshoorn, 2011). In all subsequent analyses, parameter estimates from the imputed datasets were pooled using Rubin's rules (Rubin, 1987).

#### 3.3.2 Moderated mediation analysis

The moderated mediation analysis was conducted using the bruceR package in R (Bao, 2023), following the specifications of PROCESS Model 9 (Hayes, 2017; Preacher et al., 2007). The significance of the conditional indirect effects was evaluated using a bootstrap procedure with 5,000 resamples to generate 95% confidence intervals.

#### 3.3.3 Robustness check

To assess the robustness of the findings, a non-parametric permutation test with 5,000 iterations was conducted as a validation step (Good, 2005; Anderson, 2001) using the lavaan package (Rosseel, 2012) in R. This test was used to re-examine the significance of the mediating role of social interaction and the moderating effects of social trust an d age. An effect was considered robust if its 95% confidence interval derived from the permutation test did not contain zero.

## 4 Results

### 4.1 Common method bias and missing data

The results of Harman's single-factor test suggested that common method bias was not a significant concern for this study. A logistic regression analysis was subsequently performed to examine the mechanism of missing data for the social interaction item 2 (SI2). This analysis revealed that the missingness was significantly associated with other observed covariates, thus violating the assumption of data being missing completely at random (MCAR). Consequently, multiple imputation was employed to address the missing data, wherein 20 imputed datasets were generated with a maximum of 50 iterations per imputation (*m* = 20, *maxit* = 50).

### 4.2 Descriptive statistics and correlations

The sample (*N* = 8,148) had a mean age of 51.64 years (*SD* = 17.57) and an average of 9.31 years of formal education (*SD* = 4.71). Among the participants, 45.2% were male, 66.4% resided in urban areas, and 83.9% were married. The distribution of educational attainment was as follows: no formal education (10.6%), primary school (21.6%), junior high school (28.4%), high school or vocational school (18.3%), and a bachelor's degree or higher (11.5%).

Descriptive statistics for the core variables were as follows: frequency of internet use (*M* = 3.33, *SD* = 1.67), social interaction (*M* = 2.43, *SD* = 0.84), social trust (*M* = 3.64, *SD* = 1.00), and subjective wellbeing (*M* = 3.98, *SD* = 0.82).

Correlation analyses (see Table 2 for the full matrix) indicated that frequency of internet use was positively associated with social interaction (*r* = 0.22, *p* < 0.001) and subjective wellbeing (*r* = 0.02, *p* < 0.001). Conversely, it was negatively associated with social trust (*r* = −0.09, *p* < 0.001) and age (*r* = −0.63, *p* < 0.001). Social trust was also positively associated with subjective wellbeing (*r* = 0.24, *p* < 0.001).

**Table 2:** Descriptive statistics and correlation matrix of key study variables (*N* = 8,148).

Variable	M	SD	1	2	3	4	5
1. Frequency of internet use	3.33	1.67	–				
2. Social interaction	2.43	0.84	0.22^***^	–			
3. Social trust	3.64	1.00	−0.09^***^	0.04^***^	–		
4. Subjective wellbeing	3.98	0.82	0.02^***^	0.09^***^	0.24^***^	–	
5. Age	51.64	17.57	−0.63^***^	−0.20^***^	0.13^***^	0.04^***^	–

M, Mean; SD, Standard Deviation.

^***^p < 0.001.

### 4.3 Results of the moderated mediation analysis

To test the proposed moderated mediation model, we conducted regression analyses using PROCESS Model 9 in R across the 20 multiply imputed datasets. The final parameter estimates were pooled according to Rubin's rules. The detailed results are presented in Tables 3, 4.

**Table 3:** Results of moderated mediation analysis (PROCESS Model 9, *N* = 8,148).

	Model m: social interaction			Model y: subjective wellbeing		
**Variable**	**B**	**SE**	***t***	**B**	**SE**	***t***
Gender	0.007	0.019	0.376	–0.019	0.027	–0.723
Education	0.004	0.003	1.382	0.0201^***^	0.003	7.408
Residence	0.018	0.021	0.836	0.049^*^	0.023	2.122
Marital status	–0.099^**^	0.033	–3.030	0.007	0.034	0.203
Frequency of internet use	0.089^***^	0.008	10.852	0.010	0.008	1.194
Social trust	0.053^***^	0.009	5.650	0.186^***^	0.012	15.178
Age	–0.003^**^	0.001	–3.000	0.005^***^	0.001	4.032
Internet use × social trust	0.015^**^	0.005	2.869			
Internet use × age	–0.002^***^	0.000	–5.500			
Social interaction				0.077^***^	0.013	5.868
*R* ^2^	0.066			0.075		

B, unstandardized regression coefficient; SE, standard error. The analysis was conducted on 20 multiply imputed datasets, and estimates were pooled.

^*^p < 0.05,

^**^p < 0.01,

^***^p < 0.001.

**Table 4:** Conditional indirect effects at different levels of age and social trust (*N* = 8,148).

Moderator levels		Bootstrap results for the indirect effect			
**Age**	**Social trust**	**Indirect**	**SE**	**LL 95% CI**	**UL 95% CI**
LOW	LOW	0.0087^***^	0.0019	0.0049	0.0124
LOW	MEAN	0.0098^***^	0.0020	0.0059	0.0138
LOW	HIGH	0.0110^***^	0.0022	0.0066	0.0154
MEAN	LOW	0.0057^***^	0.0013	0.0032	0.0082
MEAN	MEAN	0.0069^***^	0.0014	0.0042	0.0095
MEAN	HIGH	0.0080^***^	0.0016	0.0049	0.0111
HIGH	LOW	0.0028^**^	0.0010	0.0009	0.0047
HIGH	MEAN	0.0039^***^	0.0010	0.0021	0.0058
HIGH	HIGH	0.0051^***^	0.0012	0.0028	0.0074

Results are based on 5,000 bootstrap samples. LOW, MEAN, and HIGH represent values at one standard deviation below the mean, the mean, and one standard deviation above the mean of the respective moderator.

Indirect, conditional indirect effect; SE, standard error; LL/UL95% CI, lower/upper limit of the 95% bootstrap confidence interval.

^**^p < 0.01,

^***^p < 0.001.

**Figure 2:**
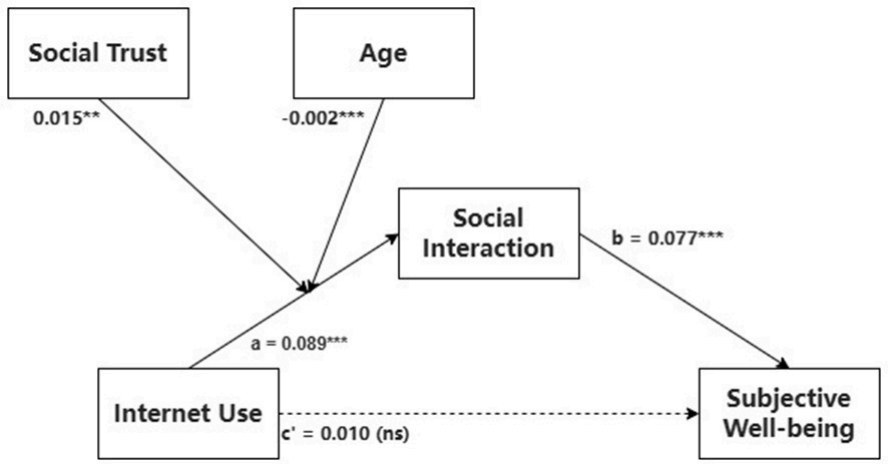
The proposed moderated mediation model with unstandardized coefficients. *Solid lines represents significant paths; dashed lines represents non-significant paths. ***p < 0.001, **p < 0.01, *p < 0.05, ns, non-significant.

The analysis began with the mediator model, where social interaction served as the outcome variable. As hypothesized, frequency of internet use was a significant and positive predictor of social interaction (*B* = 0.089, *p* < 0.001). Crucially, the results revealed significant interaction effects, indicating that both social trust and age moderated the path from internet use to social interaction (see Figure 2).

**Figure 3:**
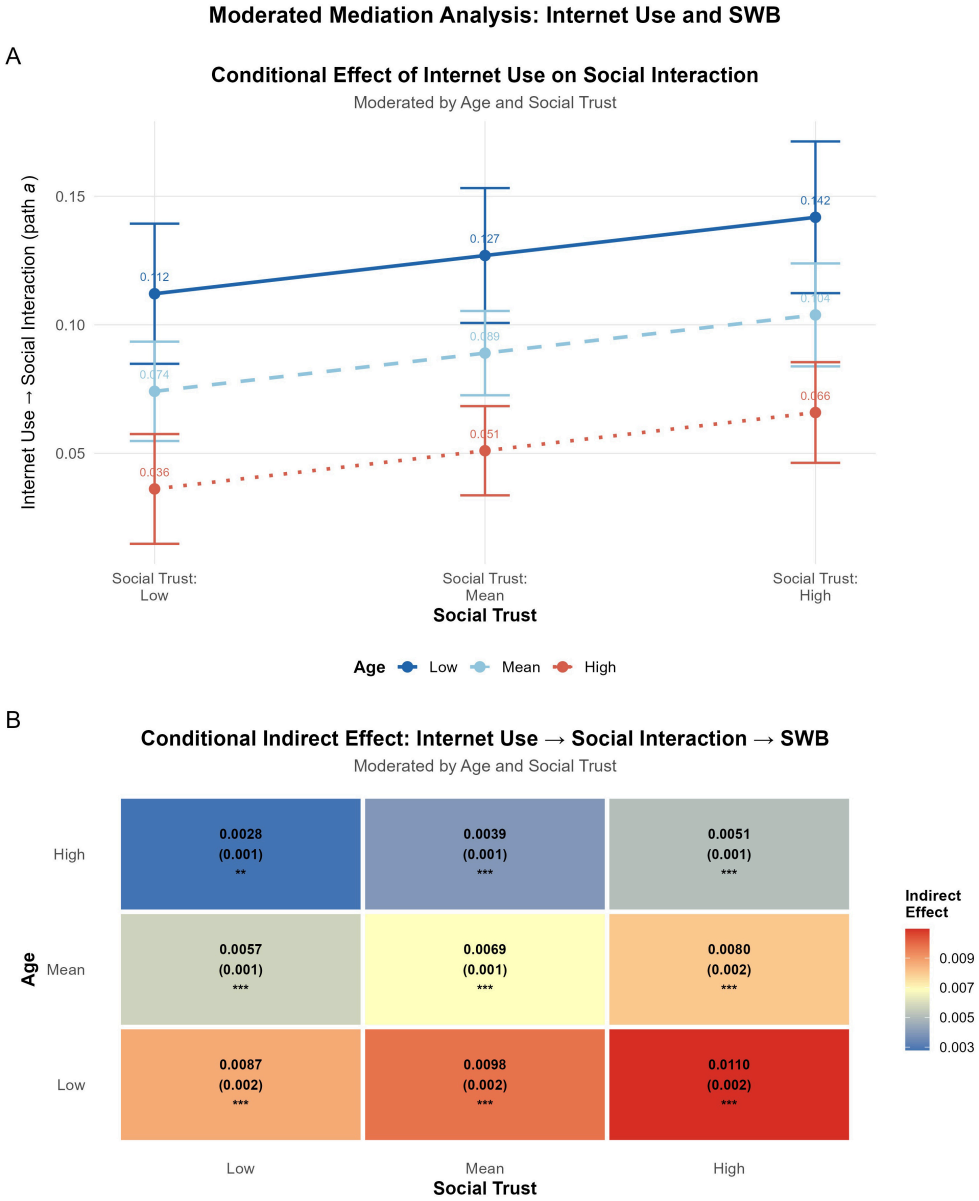
Visual representation of the moderated mediation model. **(A)** Simple slope analysis illustrating the conditional effect of Internet Use on Social Interaction, moderated by Age and Social Trust. **(B)** Heatmap of the conditional indirect effect of Internet Use on Subjective Wellbeing (via Social Interaction) across different levels of Age and Social Trust.

Regarding the moderating role of social trust, the interaction between internet use and social trust was statistically significant (*B* = 0.015, *p* < 0.01). Simple slope analysis (see Figure 3A) revealed that the positive conditional effect of internet use on social interaction was stronger for individuals with higher social trust (+1 SD) and weaker, though still positive, for those with lower social trust (–1 SD).

As for age, a significant interaction was also observed between internet use and age (*B* = −0.002, *p* < 0.001). The simple slope plot (Figure 3A) indicates that the positive relationship between internet use and social interaction attenuates as age increases, with the positive association being substantially more pronounced among younger individuals.

Next, we turned to the outcome model, with subjective wellbeing as the dependent variable. Both social interaction (*B* = 0.077, *p* < 0.001) and social trust (*B* = 0.186, *p* < 0.001) emerged as significant, positive predictors of subjective wellbeing. The direct effect of internet use frequency on subjective wellbeing was not significant (*B* = 0.010, *p*>0.05), further supporting the fully mediating role of social interaction. Among the control variables, higher educational attainment and urban residency were also associated with higher levels of subjective wellbeing.

The study's central hypothesis concerned the conditional indirect effect of internet use on subjective wellbeing, transmitted through social interaction. As detailed in Table 4, the moderated mediation analysis revealed that the indirect effect was significant across all tested combinations of age and social trust levels, with all 95% confidence intervals excluding zero.

A clear pattern emerged for the moderating role of age: the indirect effect of internet use on subjective wellbeing (via social interaction) was strongest at lower levels of age (Conditional Indirect Effect [IND] ranged from 0.0087 to 0.0110) and was substantially weaker at higher levels of age (IND ranged from 0.0028 to 0.0051). This pattern suggests that the mediating role of social interaction is conditional upon age. While internet use can enhance subjective wellbeing by fostering social interaction, the efficacy of this mechanism appears to diminish as individuals get older. This moderated relationship is visually depicted in Figure 3B.

### 4.4 Robustness check

To assess the robustness of the findings, a non-parametric permutation test with 5,000 iterations was conducted as a validation step (Good, 2005; Anderson, 2001) using the lavaan package (Rosseel, 2012) in R. This test was used to re-examine the significance of the mediating role of social interaction and the moderating effects of social trust and age. An effect was considered robust if its 95% confidence interval derived from the permutation test did not contain zero. The permutation test confirmed the robustness of the key findings; detailed results can be seen in Table 5. The effect of internet use frequency on social interaction (path *a*: *B* = 0.069, *p* < 0.001) and the effect of social interaction on subjective wellbeing (path *b*: *B* = 0.080, *p* < 0.001) were both significant. Consistent with the main analysis, the direct effect of internet use on subjective wellbeing remained non-significant (*B* = −0.005, *p* = 0.532), supporting a full mediation model wherein the association between internet use and wellbeing operates primarily through social interaction.

**Table 5:** Permutation test results for the indirect and moderation effects (*N* = 8,148).

			95% confidence interval	
Path	Estimate	SE	Lower	Upper
Indirect effect (ab)	0.012^***^	0.003	0.005	0.018
Interaction (trust)	0.015^**^	0.006	0.004	0.026
Interaction (age)	−0.002^***^	0.000	−0.003	–0.001

SE, Standard Error; CI, Confidence Interval. “Indirect effect (ab)” refers to the indirect effect of internet use on subjective wellbeing via social interaction. “Interaction (trust)” refers to the interaction between internet use and social trust. “Interaction (age)” refers to the interaction between internet use and age.

^**^p < 0.01.

^***^p < 0.001.

## 5 Discussion

These findings suggest that the association between internet use and wellbeing is not uniformly positive but may depend on how it is integrated into one's social life. Drawing on data from the 2021 Chinese General Social Survey (CGSS), this study systematically examined the pathway through which the frequency of internet use relates to subjective wellbeing, mediated by social interaction. A central focus was the dual moderating roles of social trust and age within this framework. The findings indicate that the positive link between internet use and subjective wellbeing is mediated by social interaction. More importantly, this indirect pathway appears to be conditional upon both age and social trust. Specifically, for younger individuals and those with higher levels of social trust, the potential for internet use to benefit wellbeing via social interaction is notably more pronounced. This pattern of results, suggesting a cumulative advantage for digitally adept and trusting individuals, is increasingly supported by contemporary research (Li et al., 2024; Jiang et al., 2025). Conversely, this association tends to be weaker among older individuals and those with lower social trust. These results not only support the study's theoretical hypotheses but also highlight the complex interplay among digital engagement, individual psychological attributes, and wellbeing in the contemporary era.

### 5.1 The mediating role of social interaction: from internet use to subjective wellbeing

The finding that social interaction fully mediates the relationship between internet use and subjective wellbeing underscores the role of the internet as a “social tool,” a concept consistent with social support theory (Cohen and Wills, 1985). The direct path from internet use to wellbeing was non-significant, challenging the notion of a direct technological impact and instead suggesting that the benefits of internet use are channeled primarily through enhanced social interaction. The internet transcends traditional spatio-temporal constraints, significantly lowering the costs and barriers associated with communication and relationship maintenance through platforms like social media, instant messaging, and online communities (Huang, 2010). By connecting like-minded individuals in interest-based groups and forums, it provides sustained motivation for social engagement (Lambert et al., 2013). Our finding helps to reconcile the “slew of null or contradictory findings” in the literature (Firth et al., 2024), by specifying a key mechanism.

Internet use enables residents to satisfy their leisure preferences across three major domains—socialization, entertainment, and learning—thereby enhancing wellbeing through the fulfillment of these leisure needs (Wang, 2018). Furthermore, given that the internet facilitates communication with family and friends, research indicates that its use helps older adults maintain physical and mental health while acquiring support from their social networks, including relatives and neighbors (Jiang and Chen, 2021).

These high-quality interactions fulfill fundamental needs for belongingness, provide crucial social support, and serve as a critical source of wellbeing. Social interaction, in turn, enhances subjective wellbeing through both a “main-effect pathway” (by directly fostering a sense of belonging and self-worth) and a “buffering-effect pathway” (by mitigating the psychological impact of negative life events). Ultimately, this evidence suggests that in the digital age, internet use does not directly generate happiness; rather, it nurtures it indirectly by empowering and facilitating richer, more efficient social engagement. This aligns with systematic reviews which conclude that internet use benefits wellbeing when it facilitates, rather than replaces, real-world social interaction (Ansari et al., 2024).

### 5.2 The dual moderating effects of social trust and age

A key contribution of this study is the identification of the dual moderating roles of age and social trust in the pathway linking internet use to wellbeing. Specifically, social trust significantly amplified the positive effect of internet use on social interaction, whereas age significantly attenuated this relationship. These findings can be interpreted through the combined lenses of social cognitive theory, socioemotional selectivity theory, and digital divide theory.

#### 5.2.1 The moderating role of social trust: cognitive filtering and perceived risk

The finding that social trust amplifies the positive effect of internet use on social interaction aligns closely with social cognitive theory (Bandura, 1986). Trust acts as a “psychological amplifier” or “converter,” shaping the efficiency and willingness with which individuals transform online opportunities into offline actions. This role of trust as a critical psychological pathway is directly supported by recent research (Li et al., 2024). Individuals with high levels of generalized trust are more inclined to make benevolent attributions regarding online social information. This fosters positive safety expectations and lowers defensive vigilance, making them more willing to engage with online acquaintances or accept invitations to offline events (Lu and Wei, 2023). They are better able to convert internet-based possibilities into high-quality, real-world social connections (Flanagan and Stout, 2010; Miao et al., 2025). Our finding is further strengthened by (Jiang et al. 2025), who specifically identified social trust as a significant moderator enhancing the positive effects of internet use on life satisfaction among Chinese middle-aged and older adults.

Conversely, individuals with low social trust may exhibit a heightened sensitivity to online risks (e.g., privacy violations, disinformation). Even with frequent internet use, their skepticism about others' motives may lead them to adopt defensive strategies, confining them to superficial online interactions and inhibiting the conversion of online ties into meaningful offline relationships that support wellbeing (Flanagan and Stout, 2010).

Therefore, social trust appears to shape the extent to which individuals can capitalize on the internet's social potential. This points to a “cognitive filtering effect”: high-trust individuals may focus on positive cues (e.g., support), while low-trust individuals may be more attuned to risks, which can weaken the potential benefits of internet use. This study further found that social trust indirectly relates to wellbeing through social interaction, with this association being stronger among younger people. This suggests that social trust is not merely a stable cognitive trait but a dynamic form of psychological capital that facilitates resource conversion, playing a crucial role in the digital social landscape.

#### 5.2.2 The moderating role of age: generational differences in the “use-to-interaction” conversion efficiency based on TAM

This study found that age significantly moderates the relationship between internet use and social interaction, such that the positive association of internet use with social interaction gradually diminishes with increasing age. This result can be explained not only through socioemotional selectivity theory and digital divide theory, but also further elucidated through the lens of the Technology Acceptance Model (TAM) to explore its underlying psychological mechanisms.

TAM posits that an individual's acceptance and use of a technology is jointly influenced by perceived usefulness and perceived ease of use (Davis, 1989). In the context of internet use, perceived ease of use—the degree to which an individual believes using the internet is free of effort—is likely a key psychological variable linking age to the efficiency of social interaction conversion. As individuals age, they may experience natural declines in cognitive processing speed, working memory, and visual-motor coordination (Hauk et al., 2018), while also having relatively limited exposure to new technologies (Charness and Boot, 2022). These factors collectively contribute to potentially lower perceived ease of use of the internet among older adults. Even when they do use the internet, they may feel frustrated or anxious due to operational difficulties, complex interfaces, and unfamiliar functions, making it challenging to effectively convert online behavior into positive, high-quality social interactions (e.g., initiating conversations, participating in online communities, or converting to offline meetings).

In contrast, younger people, as “digital natives,” tend to possess higher familiarity and operational confidence with various online tools and platforms in the digital environment, resulting in generally higher perceived ease of use. This may enable them to more fluently use the internet as a tool for social expansion, efficiently converting online contacts into offline interactions, and thereby obtaining more social support and emotional satisfaction. Therefore, the moderation of age on the “internet use → social interaction” pathway may essentially reflect systematic differences in perceived ease of use across age groups, which is precisely one of the core mechanisms emphasized by TAM (Cao et al., 2025). TAM provides valuable theoretical support for understanding the complex role of age in the relationship between internet use and wellbeing.

Furthermore, the observed age gradient may reflect not just a decline in efficacy, but also a shift in goals. Wellbeing gains may be optimized when internet use aligns with age-normative socioemotional objectives—a “target adjustment” perspective extending classic socioemotional selectivity theory (Carstensen, 2006).

#### 5.2.3 The conditional indirect effect: cumulative advantage for the young and trusting

Most significantly, this study reveals a synergistic interplay between age and social trust, demonstrating that the indirect effect of internet use on wellbeing—via social interaction—is strongest among young, high-trust individuals and weakest among their older, low-trust counterparts. This pattern points to a *cumulative advantage*: young, trusting individuals are optimally positioned to convert online activities into social capital and, ultimately, enhanced wellbeing. They benefit from a virtuous cycle of digital adaptability, positive social cognition, and efficient resource conversion. This cumulative advantage for the young and trusting can also be interpreted through the TAM framework: younger, high-trust individuals likely perceive the internet as both easier to use for social purposes and more useful for achieving valued social outcomes.

This finding can be understood through a developmental lens. Younger adults, despite being “digital natives,” often navigate a complex and risky online social environment. For them, social trust acts as a crucial *psychological gatekeeper*, enabling them to overcome perceived risks—such as privacy concerns and social comparison—and confidently translate online opportunities into rewarding offline social capital. In contrast, older adults with low trust face a *double disadvantage*: they are constrained not only by a relative lack of digital skills but also by a skeptical orientation toward the online world, which together impede their ability to derive wellbeing benefits from internet use.

Our findings thus uncover a more nuanced dimension of the digital divide. Digital inequality no longer stems only from gaps in physical access (the first-level divide) but also, and more critically, from disparities in *psychological access* (trust) and *capability access* (skills), which represent the second- and third-level digital divides. This multi-level perspective highlights the profound heterogeneity in how internet technologies relate to wellbeing. The “digital dividend” is not distributed uniformly; rather, it is deeply contingent upon an individual's life stage and socio-cognitive characteristics—a conclusion that aligns with broader understandings of the digital divide as encompassing not only access but also psychological and capability pathways to benefit realization (Lin et al., 2025).

## 6 Theoretical and practical implications

### 6.1 Theoretical implications

This study makes several key theoretical contributions. First, by employing a moderated mediation model, it moves beyond a simplistic direct-effects paradigm to reveal a more nuanced mechanism linking internet use to wellbeing. The finding that social trust and age jointly moderate this pathway advances our understanding of the differential susceptibility to digital effects, directly responding to scholarly calls to clarify “who benefits most from internet use” (Firth et al., 2024).

Second, the study integrates insights from digital divide theory, social support theory, and socioemotional selectivity theory into a cohesive analytical framework. This multi-pronged approach provides a more comprehensive explanation for how digital engagement interacts with life-stage priorities and cognitive orientations to shape wellbeing.

Finally, situated within China's ongoing social transformation—marked by high mobility and the rise of a “society of strangers”—the study highlights the pivotal role of generalized trust in converting online opportunities into tangible social capital. By being the first to empirically validate the combined moderating effects of age and social trust in this context, our research offers new, interdisciplinary evidence from a non-Western setting, thereby enriching global scholarship on the digital divide and its wellbeing consequences.

### 6.2 Practical implications

The findings also yield actionable insights for policy and individual practice.

These findings carry important practical implications. Given the moderating role of age, interventions should be tailored accordingly: younger users may benefit from digital literacy programs that emphasize managing social risks and information overload, while older adults may require integrated approaches that combine technical training with trust-building initiatives to bridge the digital divide.

Beyond individual-level interventions, our results also highlight the importance of cultivating social trust at the societal level. Governments and digital platforms can contribute by fostering more transparent and accountable online environments, which may in turn amplify the positive effects of internet use on social interaction and wellbeing.

Finally, individuals themselves can play an active role by intentionally translating online connections into meaningful offline relationships and participating in local community activities—practices that not only build social trust but also create a reinforcing cycle between digital engagement, social interaction, and psychological wellbeing.

## 7 Limitations and future directions

Despite its contributions, this study has several limitations that should be noted. The cross-sectional design precludes definitive causal inferences. Although our model is grounded in theory, the relationships could be bidirectional, or stem from unobserved confounders (e.g., personality traits) that might influence both internet use and wellbeing. Longitudinal or experimental designs are needed to validate the directionality of these associations.

Additionally, limitations regarding our measurement approach should be noted. First, our key independent variable—“frequency of internet use”—relies on a broad measure that does not distinguish between different types of online activities (e.g., social networking, information seeking, entertainment, or commercial use). This limits our ability to discern whether certain activities are more strongly associated with social interaction and wellbeing than others. Future research should incorporate more nuanced measures of internet use, such as time spent on specific platforms or for particular purposes, to better disentangle these heterogeneous effects.

Second, our measure of social interaction focused on frequency rather than quality. The wellbeing benefits of social engagement likely depend more on the emotional depth and supportive nature of interactions than on their sheer number. Incorporating qualitative dimensions of social ties (e.g., *strong vs. weak ties*, perceived support) would help further unpack the mediating process. Additionally, this composite scale does not differentiate between interaction modalities (e.g., face-to-face vs. online) or patterns (e.g., one-to-one vs. one-to-many communication). One-to-one communication may have different effects on wellbeing compared to one-to-many broadcasting on social media (Zhao, 2022). Future studies would benefit from distinguishing between these interaction patterns, as well as the platforms through which they occur (e.g., WeChat for private chats vs. Weibo for public posts), to better understand how digital tools shape social engagement and its psychological outcomes.

Third, all key variables were based on self-reported measures, which are susceptible to recall bias and social desirability effects. Future research could benefit from incorporating objective or behavioral data (e.g., digital trace data) to complement self-reports.

Finally, the findings are based on a Chinese sample, where cultural factors such as collectivism and relational norms may shape the observed mechanisms. Cross-cultural comparative research is needed to test the generalizability of our model and to understand how sociocultural context moderates the relationship between digital life and wellbeing.

## 8 Conclusion

This study provides evidence on the relationship between internet use and subjective wellbeing by examining the mediating role of social interaction and the joint moderating effects of social trust and age. Drawing on nationally representative data from the 2021 China General Social Survey, our findings indicate that internet use facilitates social interaction, which in turn may enhance wellbeing. Crucially, however, this indirect pathway is conditional: younger individuals and those with higher social trust appear to derive significantly greater wellbeing benefits from the social-facilitating function of the internet. These findings extend theories of the digital divide, social support, and socioemotional selectivity by suggesting that the psychosocial outcomes of internet use are not uniform. Instead, they appear to be systematically shaped by individual differences in age and social trust. The study also highlights the unique importance of social trust in translating internet use into meaningful social connections and, ultimately, enhanced wellbeing, particularly within the Chinese context. From a practical standpoint, our results advocate for tailored strategies—such as promoting digital literacy among youth, encouraging balanced online-offline engagement for the middle-aged, and providing targeted support for older adults. At a macro level, fostering a transparent and trustworthy digital ecosystem is essential for strengthening societal trust and maximizing the wellbeing benefits of the internet.

## Data Availability

Publicly available datasets were analyzed in this study. This data can be found here: http://cgss.ruc.edu.cn.
