# Supplementary material for: Internet benefits are not one-size-fits-all: age and trust shape digital wellbeing gains
Source: Front Psychol. 2026 Apr 10;17:1736005. doi: 10.3389/fpsyg.2026.1736005 (PMC13106340; doi:10.3389/fpsyg.2026.1736005)
Supplement: Supplementary file 1 [file Data_Sheet_1.pdf]

Supplementary Material

1 SUPPLEMENTARY TABLES

1.1 Variable Name Correspondence

Table S0 presents the correspondence between variable names used in the main manuscript and those in the original dataset and analysis code.

Table S1. Correspondence between manuscript and dataset variable names

Main Manuscript	Dataset/Code	Description
Internet Use (IU)	IUA	Frequency of Internet Use
Social Interaction (SI)	SI	Social Interaction Frequency
Social Trust (ST)	ST	Social Trust
Subjective Well-being (SWB)	HP	Subjective Well-being (Happiness)
Age	AGE	Age in years
Education	EDU_years	Years of Education
Gender	SEX	Gender (0=Female, 1=Male)
Marital Status	MARI	Marital Status (0=Unmarried, 1=Married)
Residence	UOR	Urban or Rural Residence

Note. The main manuscript uses conceptually clear variable names for readability, while the supplementary materials and analysis code retain the original dataset variable names to ensure reproducibility. All statistical analyses were conducted using the dataset variable names shown in the middle column.

1.2 Table S1. Descriptive Statistics and Correlation Matrix

Table S1 presents means, standard deviations, and Pearson correlation coefficients for all study variables. **Variable names correspond to the dataset/code column in Table S0.**

Table S2. Descriptive statistics and correlation matrix of all variables (dataset variable names)

Variable	M±SD	IUA	SI	ST	HP	AGE	EDU	SEX	MARI	U[illegible]
IUA	3.33±1.67	—								
SI	2.43±0.84	0.22***	—							
ST	3.64±1.00	-0.09***	0.04***	—						
HP	3.98±0.82	0.02***	0.09***	0.24***	—					
AGE	51.64±17.57	-0.63***	-0.20***	0.13***	0.04***	—				
EDU_years	9.31±4.71	0.55***	0.17***	-0.02***	0.09***	-0.54***	—			
SEX	0.45±0.50	0.01	0.01**	0.05***	0.02***	0.03***	0.13***	—		
MARI	0.84±0.37	-0.26***	-0.14***	0.04***	0.01	0.52***	-0.35***	-0.07***	—	
UOR	0.66±0.47	0.24***	0.07***	-0.06***	0.05***	-0.12***	0.30***	-0.03***	-0.07***	[illegible]

Note. **p* ¡ .05, ***p* ¡ .01, ****p* ¡ .001. Variable names correspond to the dataset/code names (see Table S0 for correspondence with main manuscript names).
Variable Definitions:
IUA (Internet Use in manuscript) = Frequency of Internet Use (1 = *Never*; 5 = *Very frequently*). Measured by: ”In the past year, how often did you use the Internet (including mobile access)?”
SI = Social Interaction Frequency. Composite variable based on four items (SI1-SI4) asking about frequency of social activities with relatives, friends, neighbors, and other acquaintances (1 = *Never*; 5 = *Almost every day*).
ST = Social Trust (1 = *Strongly disagree*; 5 = *Strongly agree*). Measured by: ”Generally speaking, do you think most people in society can be trusted?”
HP (Subjective Well-being in manuscript) = Happiness (1 = *Very unhappy*; 5 = *Very happy*). Measured by: ”Generally speaking, do you feel your life is happy?”
AGE = Age in years, calculated from year of birth.
EDU_years (Education in manuscript) = Years of Education. Converted from categorical levels: 0 = No formal education; 2 = Private tutoring; 6 = Primary school; 9 = Junior high; 12 = High school/Technical; 15 = Junior college; 16 = Bachelor’s; 19 = Postgraduate.
SEX (Gender in manuscript) = Gender (0 = Female; 1 = Male).
MARI (Marital Status in manuscript) = Marital Status (0 = Unmarried; 1 = Married).
UOR (Residence in manuscript) = Urban or Rural Residence (0 = Rural; 1 = Urban).

2 SUPPLEMENTARY FIGURES

2.1 Figure S1. Missing Data Pattern Analysis

Figure S1 displays the percentage of missing data for each variable in the original dataset before multiple imputation.

2.2 Figure S2. Multiple Imputation Convergence Diagnostics

Figures S2A and S2B present trace plots for assessing the convergence of the multiple imputation algorithm across 50 iterations for 20 imputed datasets (m = 20).

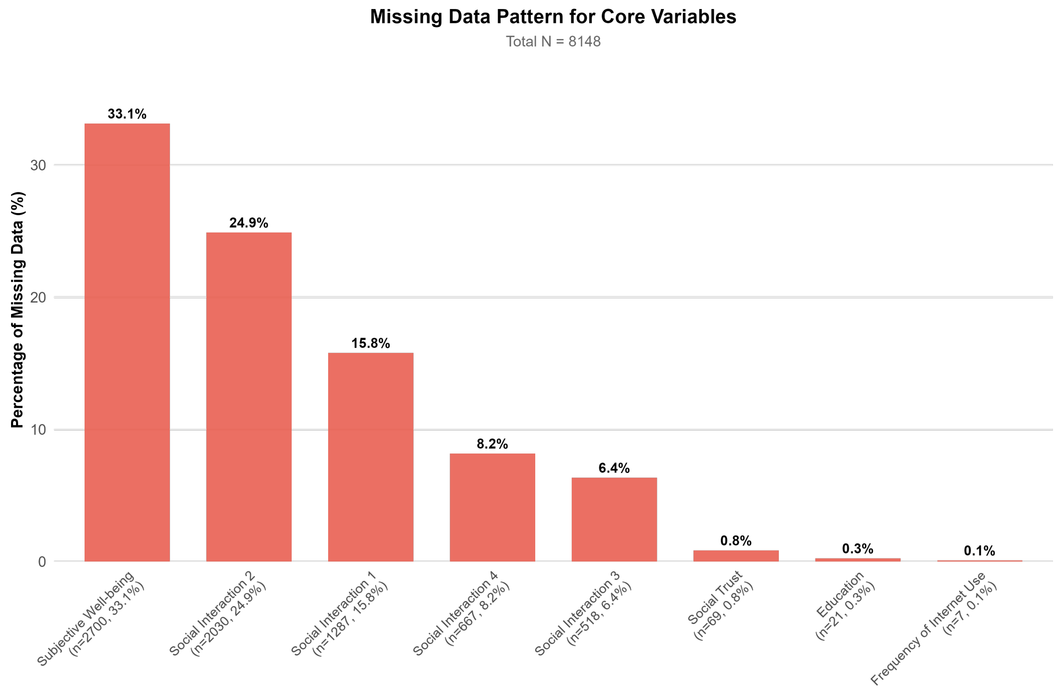

Figure S1. Missing data pattern across all study variables. The bar chart shows the percentage of missing values for each variable. Missing rates ranged from 0.1% to 33.1%, with the highest missingness observed in Education and Subjective Well-being , followed by Social Interaction items (SI1-SI4). Multiple imputation using the MICE algorithm with 20 imputed datasets (m = 20) was applied to handle missing data.

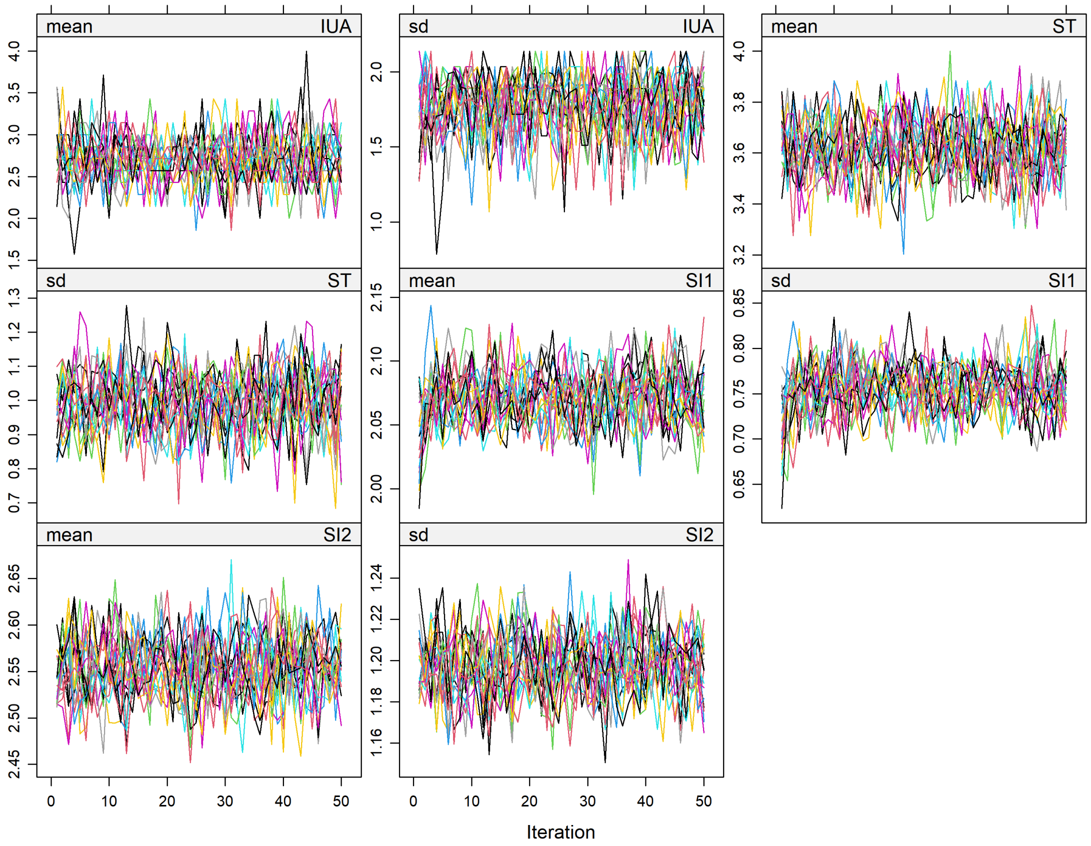

Figure S2. Convergence diagnostic plots (Part 1). Trace plots showing the mean and standard deviation of imputed values across 50 iterations for Frequency of Internet Use (IUA), Social Trust (ST), and Social Interaction items (SI1-SI2). Each colored line represents one of the 20 imputed datasets. Stable convergence is indicated by the overlapping and stable traces across iterations, with no systematic trends or divergence.

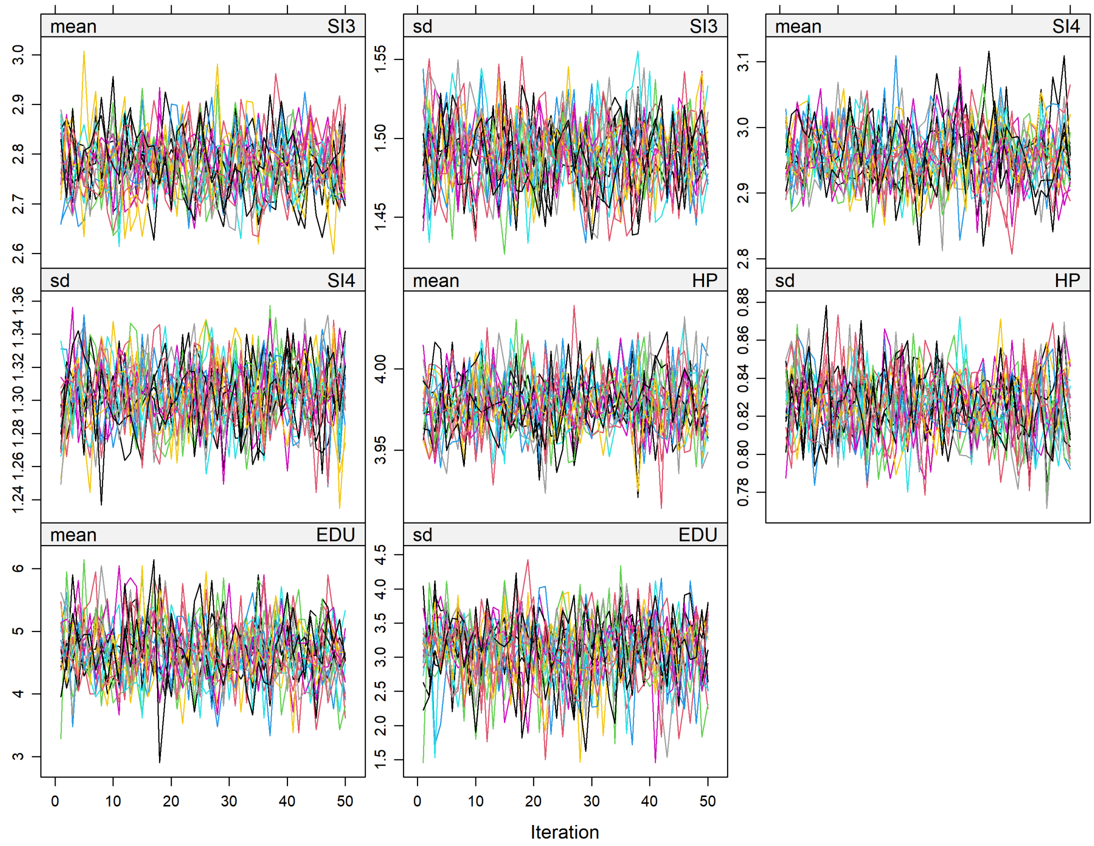

Figure S3. Convergence diagnostic plots (Part 2). Trace plots showing the mean and standard deviation of imputed values across 50 iterations for Social Interaction items (SI3-SI4), Subjective Well-being (HP), and Education (EDU). The stable patterns with minimal fluctuation after initial iterations confirm successful convergence of the MICE imputation algorithm for all variables.

3 SUPPLEMENTARY CODE

All analysis code is provided below. The code is fully reproducible and includes detailed comments. **Variable names used in the code correspond to the dataset names in Table S0.**

3.1 Code S1: PROCESS Moderated Mediation Analysis

This script implements moderated mediation analysis using the `bruceR` package's PROCESS function (Hayes, 2017). The analysis tests the indirect effect of Internet Use Frequency (IUA) on Subjective Well-being (HP) through Social Interaction (SI), with Social Trust (ST) and Age (AGE) as moderators on the X→M path.

Key features:

- Analyzes 20 multiply imputed datasets (N = 8,148 per dataset)
- Uses 5,000 bootstrap samples per imputation for confidence intervals
- Unique random seed per imputation (123+i) ensures reproducibility
- Controls for sex, education, marital status, and urban/rural residence
- Saves both R objects (.rds) and text reports (.txt) for each imputation

```
# ==============================================================================
# Code S1: PROCESS Moderated Mediation Analysis
# Model: PROCESS Model 9 (Hayes, 2017)
# N = 8,148 | Bootstrap iterations = 5,000
# ==============================================================================

library(bruceR)

# Configuration
OUT_DIR_PROCESS <- "output/process_results"
OUT_DIR_REPORTS <- "output/process_reports"
dir.create(OUT_DIR_PROCESS, recursive = TRUE, showWarnings = FALSE)
dir.create(OUT_DIR_REPORTS, recursive = TRUE, showWarnings = FALSE)

# Define variables
covariates_vec <- c("SEX", "EDU_years", "UOR", "MARI")  # Control variables
mods_vec       <- c("ST", "AGE")                        # Moderators

# Function: PROCESS Analysis for Single Imputed Dataset
analyze_imputation <- function(i, imp_data_list, covariates_vec, mods_vec) {
  set.seed(123 + i)  # Unique seed per imputation

  # Run PROCESS Model 9
  # X (IUA) → M (SI) → Y (HP)
  # Moderators (ST, AGE) on X→M path
```

```
  pro_i <- PROCESS(
    data  = imp_data_list[[i]],
    y     = "HP",              # Outcome: Subjective Well-being
    x     = "IUA",             # Predictor: Internet Use Frequency
    meds  = "SI",              # Mediator: Social Interaction
    mods  = mods_vec,          # Moderators: Social Trust & Age
    covs  = covariates_vec,    # Control variables
    mod.path = "x-m",          # Moderation on X→M path
    ci    = "boot",            # Bootstrap CI
    nsim  = 5000,              # 5000 bootstrap samples
    seed  = 123 + i
  )

  # Save results
  saveRDS(pro_i,
          file = file.path(OUT_DIR_PROCESS,
                          sprintf("process_imp%02d.rds", i)))

  # Save text report
  report_file <- file.path(OUT_DIR_REPORTS,
                          sprintf("report_imp%02d.txt", i))
  capture.output({
    cat(sprintf("========== PROCESS Report: Imputation %d ==========\n", i))
    print(pro_i)
  }, file = report_file)

  cat(sprintf("✓ Completed imputation %d\n", i))
  return(pro_i)
}

# Main Analysis: Loop Through 20 Imputed Datasets
process_results <- list()

for (i in 1:20) {
  cat(sprintf("\n--- Analyzing Imputation %d/20 ---\n", i))
  process_results[[i]] <- analyze_imputation(i, imp_data_list,
                                             covariates_vec, mods_vec)
}

cat("\n✓ All 20 imputations completed!\n")

# ==============================================================================
# Notes:
# - Input data (imp_data_list) not included in this script
# - Expected variables: HP, IUA, SI, ST, AGE, SEX, EDU_years, UOR, MARI
```

```
# - Each imputation uses unique seed (123+i) for reproducible bootstrap
# - Results saved as both RDS objects and text reports
# ============================================================================
```

3.2 Code S2: Permutation Test for Moderated Mediation Effects

This script conducts permutation-based inference to validate the mediation and moderation effects. It uses structural equation modeling via `lavaan` and pools results across imputations using Rubin's rules.

Key features:

- Performs 5,000 permutations per imputation (100,000 total samples)
- Permutes the predictor (IUA) while preserving data structure
- Pools point estimates and variances using Rubin's (1987) formulas
- Computes permutation p-values from stacked null distribution
- Provides non-parametric validation of bootstrap-based inference

```
# ============================================================================
# Code S2: Permutation Test for Moderated Mediation Effects
# Method: 5,000 permutations per imputation, Rubin's pooling
# N = 8,148
# ============================================================================

suppressPackageStartupMessages({
  library(lavaan)
  library(dplyr)
})

# Configuration
OUT_DIR_PERM <- "output/permutation_results"
dir.create(OUT_DIR_PERM, recursive = TRUE, showWarnings = FALSE)

# Helper Functions: Rubin's Pooling Rules
rubin_pool <- function(est_mat, var_mat) {
  m <- nrow(est_mat)
  qbar <- colMeans(est_mat, na.rm = TRUE)          # Pooled estimate
  ubar <- colMeans(var_mat, na.rm = TRUE)          # Within-imputation variance
  b    <- apply(est_mat, 2, var, na.rm = TRUE)     # Between-imputation variance
  Tvar <- ubar + (1 + 1/m) * b                     # Total variance
  se   <- sqrt(Tvar)

  # Barnard-Rubin degrees of freedom
  r  <- (1 + 1/m) * b / ubar
  df <- (m - 1) * (1 + 1/r)^2

  list(qbar = qbar, se = se, df = df, Tvar = Tvar)
}

t_crit <- function(df, alpha = 0.05) {
  qt(1 - alpha/2, df = df)  # Two-tailed t critical value
```

```
}

# Lavaan Model: Moderated Mediation
mod_model <- '
  # Mediator equation: IUA → SI (moderated by ST and AGE)
  SI ~ a*IUA + ST + AGE + SEX + EDU_years + MARI + UOR
  SI ~ intST*IUA_ST + intAGE*IUA_AGE

  # Outcome equation: SI → HP (with direct effect of IUA)
  HP ~ b*SI + c*IUA + SEX + AGE + EDU_years + MARI + UOR

  # Indirect effect
  ab := a*b

  # Moderation effects
  int_ST_effect := intST
  int_AGE_effect := intAGE
'

# Function: Fit Model + Permutation for One Imputation
fit_and_permute_one <- function(data, model, n_perm = 5000) {
  # Prepare interaction terms
  data$IUA_ST  <- data$IUA * data$ST
  data$IUA_AGE <- data$IUA * data$AGE

  # Original fit (observed data)
  fit0 <- sem(model, data = data, estimator = "MLR")
  est0 <- parameterEstimates(fit0, se = TRUE)

  # Extract estimates for: ab, intST, intAGE
  pick <- function(df) {
    df$est[match(c("ab", "intST", "intAGE"), df$label)]
  }
  pick_se2 <- function(df) {
    (df$se[match(c("ab", "intST", "intAGE"), df$label)])^2
  }

  q0 <- pick(est0)
  u0 <- pick_se2(est0)
  names(q0) <- names(u0) <- c("ab", "intST", "intAGE")

  # Permutation: shuffle IUA, recompute interactions
  perm_mat <- matrix(NA_real_, nrow = n_perm, ncol = 3,
                     dimnames = list(NULL, c("ab", "intST", "intAGE")))
```

```
  for (i in 1:n_perm) {
    datp <- data
    datp$IUA     <- sample(data$IUA)       # Permute predictor
    datp$IUA_ST  <- datp$IUA * datp$ST     # Recompute interactions
    datp$IUA_AGE <- datp$IUA * datp$AGE

    fitp <- try(sem(model, data = datp, estimator = "MLR"), silent = TRUE)
    if (!inherits(fitp, "try-error")) {
      estp <- parameterEstimates(fitp, se = FALSE)
      perm_mat[i, ] <- pick(estp)
    }
  }

  list(est = q0, var = u0, perm = perm_mat)
}

# Function: Run Permutation Test Across All Imputations
run_mi_permutation <- function(imp_data_list, model, n_perm = 5000) {

  all_est  <- list()
  all_var  <- list()
  all_perm <- list(ab = NULL, intST = NULL, intAGE = NULL)

  # Loop through imputations
  for (j in seq_along(imp_data_list)) {
    cat(sprintf("Permutation test: imputation %d/%d\n",
                j, length(imp_data_list)))

    one <- fit_and_permute_one(as.data.frame(imp_data_list[[j]]),
                               model, n_perm)

    all_est[[j]] <- one$est
    all_var[[j]] <- one$var

    # Stack permutation samples across imputations
    all_perm$ab     <- c(all_perm$ab,     one$perm[, "ab"])
    all_perm$intST  <- c(all_perm$intST,  one$perm[, "intST"])
    all_perm$intAGE <- c(all_perm$intAGE, one$perm[, "intAGE"])
  }

  # Combine estimates across imputations
  est_mat <- do.call(rbind, all_est)  # m × 3
  var_mat <- do.call(rbind, all_var)  # m × 3

  # Rubin's pooling
```

```
  rub  <- rubin_pool(est_mat, var_mat)
  qbar <- rub$qbar
  se   <- rub$se
  df   <- rub$df

  # 95% Confidence intervals
  tval <- t_crit(df)
  ci_l <- qbar - tval * se
  ci_u <- qbar + tval * se

  # Permutation p-values (two-tailed)
  perm_p <- c(
    ab     = mean(abs(all_perm$ab)     >= abs(qbar["ab"]),     na.rm = TRUE),
    intST  = mean(abs(all_perm$intST)  >= abs(qbar["intST"]),  na.rm = TRUE),
    intAGE = mean(abs(all_perm$intAGE) >= abs(qbar["intAGE"]), na.rm = TRUE)
  )

  # Results table
  results <- data.frame(
    Path           = c("ab", "intST", "intAGE"),
    Estimate       = as.numeric(qbar[c("ab", "intST", "intAGE")]),
    SE             = as.numeric(se[c("ab", "intST", "intAGE")]),
    df             = as.numeric(df[c("ab", "intST", "intAGE")]),
    CI_Lower       = as.numeric(ci_l[c("ab", "intST", "intAGE")]),
    CI_Upper       = as.numeric(ci_u[c("ab", "intST", "intAGE")]),
    Permutation_p  = as.numeric(perm_p[c("ab", "intST", "intAGE")])
  )

  # Save results
  saveRDS(all_perm, file.path(OUT_DIR_PERM, "permutation_samples.rds"))
  write.csv(results, file.path(OUT_DIR_PERM, "permutation_results.csv"),
            row.names = FALSE)

  cat("\n✓ Permutation test completed!\n")
  return(results)
}

# Main Analysis
set.seed(20250818)

# Load imputed data (not included in this script)
# imp_data_list <- readRDS("data/imp_data_list.rds")

perm_results <- run_mi_permutation(imp_data_list,
                                   model = mod_model,
```

```
                                  n_perm = 5000)

print(perm_results)

# ==============================================================================
# Notes:
# - Input data (imp_data_list) not included in this script
# - Expected variables: HP, IUA, SI, ST, AGE, SEX, EDU_years, UOR, MARI
# - Total permutations: 5,000 × 20 imputations = 100,000 samples
# - Results pooled using Rubin's rules for multiple imputation
# - Permutation p-values computed from stacked null distribution
# ==============================================================================
```

Note. Input data (`imp_data_list`) contains 20 multiply imputed datasets generated using the `mice` package (v3.16.0) with m=20 imputations and maxit=50 iterations. The imputed datasets are not included in this supplementary material to protect participant privacy. Complete executable code and de-identified data are available from the corresponding author upon reasonable request, subject to ethical approval.
